# Development of a risk assessment model for cardiac injury in patients newly diagnosed with acute myeloid leukemia based on a multicenter, real-world analysis in China

**DOI:** 10.1186/s12885-024-11847-0

**Published:** 2024-01-25

**Authors:** Linlu Ma, Qian Wang, Xinqi Li, Yufeng Shang, Nan Zhang, Jinxian Wu, Yuxing Liang, Guopeng Chen, Yuxin Tan, Xiaoyan Liu, Guolin Yuan, Fuling Zhou

**Affiliations:** 1 Department of Hematology, Zhongnan Hospital of Wuhan University, Wuhan University, Wuhan, Hubei China; 2 Department of Hematology, The First Affiliated Hospital of Zhengzhou University, Zhengzhou, Henan China; 3 Department of Hematology, Xiangyang Central Hospital, The Affiliated Hospital of Hubei University of Arts and Science, Xiangyang, Hubei China

**Keywords:** Acute myeloid leukemia, Risk factor, Prediction model, Inflammation, Cardiac injury

## Abstract

**Background:**

Studies have revealed that acute myeloid leukemia (AML) patients are prone to combined cardiac injury. We aimed to identify hematological risk factors associated with cardiac injury in newly diagnosed AML patients before chemotherapy and develop a personalized predictive model.

**Methods:**

The population baseline, blood test, electrocardiogram, echocardiograph, and genetic and cytogenetic data were collected from newly diagnosed AML patients. The data were subdivided into training and validation cohorts. The independent risk factors were explored by univariate and multivariate logistic regression analysis respectively, and data dimension reduction and variable selection were performed using the least absolute shrinkage and selection operator (LASSO) regression models. The nomogram was generated and the reliability and generalizability were verified by receiver operating characteristic (ROC) curves, the area under the curve (AUC) and calibration curves in an external validation cohort.

**Results:**

Finally, 499 AML patients were included. After univariate logistic regression, LASSO regression and multivariate logistic regression analysis, abnormal NT-proBNP, NPM1 mutation, WBC, and RBC were independent risk factors for cardiac injury in AML patients (all *P* < 0.05). The nomogram was constructed based on the above four variables with high accuracy. The area under the curve was 0.742, 0.750, and 0.706 in the training, internal validation, and external validation cohort, respectively. The calibration curve indicated that the model has good testing capability. The Kaplan-Meier curve showed that the higher the risk of combined cardiac injury in AML patients, the lower their probability of survival.

**Conclusions:**

This prediction nomogram identifies hematological risk factors associated with cardiac injury in newly diagnosed AML patients and can help hematologists identify the risk and provide precise treatment options.

## Background

Cancer and cardiovascular disease (CVD) are the two most common diseases in the world with a higher mortality rate [1]. Leukemia is a life-threatening hematological cancer, with 474,519 new cases and 311,594 deaths worldwide in 2020 [2]. Acute myeloid leukemia (AML) is the most common acute leukemia in adults, with a 5-year survival rate of less than 40% [3]. From 1990 to 2017, the global number of disability-adjusted life years caused by AML increased by 56.14% [4]. Especially for the AML cases over 60 years old, accounting for the vast majority of AML patients, the 5-year overall survival (OS) is only 10–20% [5].

Greater overlap between cancer and CVD is observed due to an aging population and the sharing of common risk factors and biological pathways [6]. The common risk factors of both disease entities [7] include tobacco smoking, obesity, unhealthy diet, hypertension, diabetes, etc. The potential mechanisms contributing to this overlap include chronic inflammation, oxidative stress, a prothrombotic state, metabolic derangements, genetic predisposition, and clonal hematopoiesis of indeterminate potential (CHIP) [8]. Additionally, autopsies conducted on individuals who succumbed to acute leukemia (AL) revealed that a considerable proportion, up to 37%, exhibited leukemic infiltrates within the cardiac structures, including the walls of cardiac chambers, pericardia and subepicardial adipose tissue [9–11]. Furthermore, the occurrence of leukemic cardiac infiltration was notably higher in AML compared to acute lymphoblastic leukemia (ALL) [12]. It is worth noting that while certain leukemia patients were admitted to the hospital due to cardiac manifestations as their initial [13–15] or accompanying symptoms [16–18], the reported incidence of cardiac injury in the conducted studies may only represent a fraction of the actual prevalence, primarily due to the absence of early cardiac monitoring [19].

Hence, it is postulated that leukemia per se induces some cardiac injury through the release of excessive cytokines or infiltration of leukemic cells, even before the administration of anti-tumor medications. Our team conducted a cardiac assessment of newly diagnosed AL patients in the Chinese population and found that the myocardial enzyme levels in AL patients were significantly higher than those in healthy controls [20]. Consequently, we intend to investigate additional hematological risk factors linked to cardiac injury in newly diagnosed AML patients, with the ultimate goal of constructing a prediction model that would assist hematologists in identifying varying levels of risk and offering precise treatment options for individual patients.

## Materials and methods

### Participants of inclusion

Data for this study were divided into three data sets from two medical centers. We analyzed the information of newly diagnosed AML patients between January 2017 and December 2022 at Wuhan University Zhongnan Hospital and Xiangyang City Centre Hospital. AML patients from Wuhan University Zhongnan Hospital were randomly divided into a training set and a testing set according to a ratio of 7:3, and those from Xiangyang City Centre Hospital were used as an external validation set. This study complied with the ethical guidelines of the Declaration of Helsinki and was approved by the ethics review committee of Wuhan University Zhongnan Hospital.

All included patients were diagnosed with AML according to the 2016 revision to the World Health Organization classification of myeloid neoplasms and acute leukemia [21] and did not receive chemotherapy. Patients younger than 18 years old, with prior or concurrent malignancy, with a known history of cardiomyopathy, coronary artery disease (CAD) or heart failure (HF) were excluded from this study. The cases without cardiac biomarkers, including values of high-sensitivity troponin I (hs-TNI) or creatinine kinase-myocardial band (CK-MB) or electrical abnormal markers, were excluded.

### Definition of cardiac injury and outcomes

Previous studies have defined cardiac injury based on cardiac biomarkers obtained through blood sampling and the abnormalities on ECG [22–24]. Combined with literature and clinical experience, cardiac injury was defined when the following abnormalities were present at the same time: (1) cardiac biomarkers (abnormal CK-MB or hs-TNI, above the 99th-percentile upper reference limit); (2) electrical abnormal markers (cardiac arrhythmias, such as: atrial flutter/fibrillation, supraventricular tachycardia, ventricular tachycardia and ventricular fibrillation, bundle branch block, T wave flattening/inversion, ST-segment elevation/depression and QT interval prolongation were first detected or recently developed on ECG). In addition, we divided these newly diagnosed AML patients into two groups according to whether they had concomitant cardiac injury, assessed factors associated with cardiac injury, and identified patients at high risk of cardiac injury to determine appropriate prevention strategies.

#### Risk factors exploration and nomogram establishment

Gender, age, cardiovascular risk factors (history of smoking, hypertension, and diabetes), laboratory tests: complete blood count, myocardial enzymes, N-terminal pro-B-type natriuretic peptide (NT-proBNP), hs-TNI, bone marrow (BM) manifestation, molecular genetics, cytogenetics, ECG and echocardiogram data were all obtained from electronic medical records with standardized data collection forms. The data were reviewed by a team of trained physicians who also followed up the patients’ survival status and survival time.

Univariate logistic regression was used to identify independent variables, and nonzero coefficients were identified by the least absolute shrinkage and selection operator (LASSO) regression analysis. Then, multivariate logistic regression was performed to acquire variables. A nomogram prediction model was developed, and its efficacy was assessed through internal and external validation in the testing set and external validation set respectively. The predictive accuracy of the nomograms was assessed by using receiver operating characteristic curve (ROC) analysis and calculating the area under the curve (AUC). Calibration curves were used to compare the agreement of the predicted and actual probabilities of the nomogram. We calculated all risk scores from the nomogram and classified them as low and high risk, respectively. Finally, we also assessed the prognosis by risk stratification.

### Statistical analysis

All statistical analyzes were performed using IBM SPSS version 20.0 and R version 4.2.2. Continuous variables were reported as median with interquartile ranges (IQR), while categorical variables were reported as numbers with proportions. Patient characteristics were compared using the chi-square test or Fisher’s exact test for categorical variables, and the Mann-Whitney U test for continuous variables using IBM SPSS statistics software. The survival rate of AML patients with or without cardiac injury was analyzed by Kaplan-Meier (KM) curve. Univariate logistic regression, imputation of a small amount of missing data, the LASSO regression and multivariate logistic regression were performed by R to screen meaningful variables to develop a predictive model. Nomograms, calibration curves and ROC were performed or plotted using R version 4.2.2 ultimately. All reported p-values were two-sided with a significance level of 0.05.

## Results

### Basic characteristics of patients

**Fig. 1:**
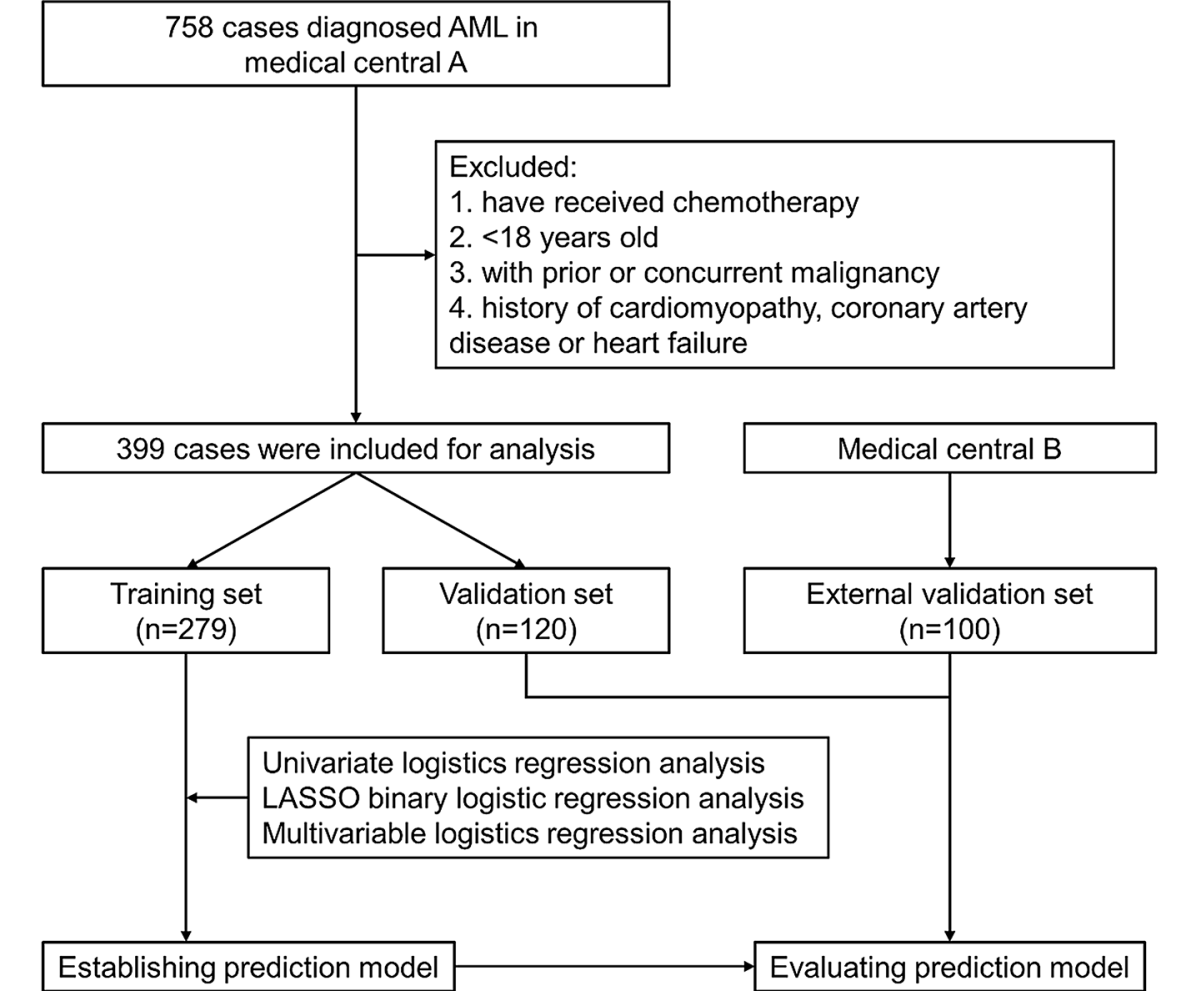
The flow chart of analysis in this study

Finally, 499 patients with newly diagnosed AML from two medical centers, including 272 males and 227 females, were included in this study. Data from 399 patients from Wuhan University Zhongnan Hospital were used for the training and testing sets, and data from 100 patients from Xiangyang City Centre Hospital were used for the external validation set. The analysis flow chart of this study was shown in Fig. 1.

### Comparison of basic characteristics between AML patients with or without cardiac injury

**Fig. 2:**
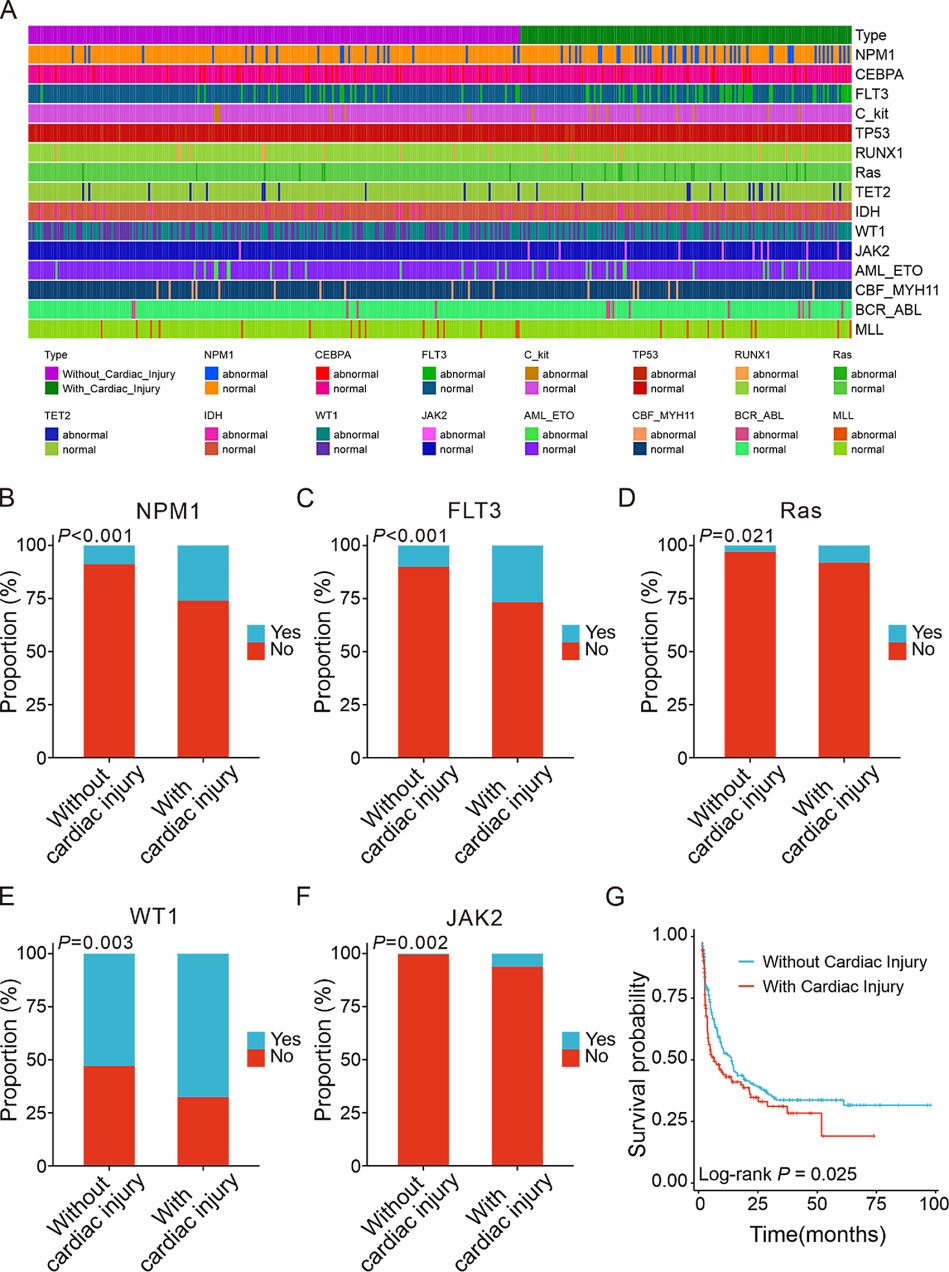
Effects of cardiac injury status on different molecular characteristics and overall survival in AML patients. (**A**). Heatmap to visualize the association of cardiac injury status and clinical characteristics. (**B**)-(**F**). Histograms of the proportion of NPM1, FLT3, Ras, WT1 and JAK2 gene mutations in the state of concomitant heart injury or not. (**G**). Kaplan-Meier curves of cardiac injury for OS in newly diagnosed AML patient. OS, overall survival

The cohort of newly diagnosed AML patients was divided into two groups based on the occurrence of cardiac injury for characteristic assessment. The clinical, molecular and biochemical characteristics of the above two groups of patients were summarized in detail below (Table 1). There were no significant differences in gender, age, cardiovascular risk factors (the smoking history, hypertension history and diabetes history) between the two groups. The AML patients with cardiac injury had higher WBC counts (*P* < 0.001) and marrow blasts (*P* = 0.007), and a higher percentage of gene mutations, such as NPM1 (*P* < 0.001), FLT3 (*P* < 0.001), Ras (*P* = 0.021), WT1 (*P* = 0.003) and JAK2 (*P* = 0.002), when compared to the patients without cardiac injury. Moreover, the patients with cardiac injury were more often diagnosed with M5 (*P* = 0.004), but less diagnosed with M1 compared with other subgroups (*P* = 0.026). However, no statistical differences were observed between the two groups in terms of cytogenetics. In addition, AML patients with cardiac injury had a higher rate of abnormal NT-proBNP (*P* < 0.001) and lower EF values. Finally, to determine the prognostic effect of combined cardiac injury on AML patients, we performed a KM analysis of the two groups, and the KM curves showed that patients with newly diagnosed AML combined with cardiac injury had a poorer prognosis (*P* = 0.025), see Fig. 2.

**Table 1:** Basic characteristics of newly diagnosed AML patients with or without cardiac injury

Characteristics	Total (n = 399) No. (%) / Median (IQR)	Comparison		
		With cardiac injury (n = 161) No. (%) / Median (IQR)	Without cardiac injury (n = 238) No. (%) / Median (IQR)	*P*
Gender				0.863
Male	216(54.1)	88(54.7)	128(53.8)	
Female	183(45.9)	73(45.3)	110(46.2)	
Age (years)				0.309
Age < 60	218(54.6)	83(51.6)	135(56.7)	
Age ≥ 60	181(45.4)	78(48.4)	103(43.3)	
Smoking history	67(16.8)	27(16.8)	40(16.8)	0.992
History of hypertension	67(16.8)	28(17.4)	39(16.4)	0.792
History of diabetes	29(7.3)	16(9.9)	13(5.5)	0.091
WBC (10^9^/L)	10.14(2.2-37.13)	24.6(5.87–77.2)	5.27(1.72-15.0425)	< 0.001
RBC (10^12^/L)	2.19(1.7–2.62)	2.32(1.83–2.87)	2.12(1.65–2.56)	0.002
Hb (g/L)	70(56.4–85.8)	70(59.35–87.4)	69.55(54.6-84.85)	0.152
PLT (10^9^/L)	38(21–73)	33(19-77.5)	39(21–73)	0.602
BM blast ratio (%)	59.5(35.5–80.5)	64(37-83.25)	57(33.375-76)	0.007
FAB subtypes				
M1	32(8)	7(4.3)	25(10.5)	0.026
M2	176(44.1)	66(41.0)	110(46.2)	0.302
M3	52(13)	19(11.8)	33(13.9)	0.548
M4	22(5.5)	8(5.0)	14(5.9)	0.695
M5	72(18)	40(24.8)	32(13.4)	0.004
M6	5(1.3)	3(1.9)	2(0.8)	0.658
Unkown	40(10)	18(11.2)	22(9.2)	0.527
Gene mutations				
NPM1	63(15.8)	42(26.1)	21(8.8)	< 0.001
CEBPA	42(10.5)	17(10.6)	25(10.5)	0.986
FLT3	67(16.8)	43(26.7)	24(10.1)	< 0.001
C-kit	15(3.8)	9(5.6)	6(2.5)	0.114
TP53	24(6.0)	12(7.5)	12(5.0)	0.320
RUNX1	17(4.3)	7(4.3)	10(4.2)	0.943
Ras	20(5.0)	13(8.1)	7(2.9)	0.021
TET2	26(6.5)	14(8.7)	12(5.0)	0.147
IDH	37(9.3)	18(11.2)	19(8.0)	0.280
WT1	235(58.9)	109(67.7)	126(52.9)	0.003
JAK2	11(2.8)	10(6.2)	1(0.4)	0.002^#^
AML1-ETO	29(7.3)	14(8.7)	15(6.3)	0.366
CBFB-MYH11	17(4.3)	6(3.7)	11(4.6)	0.664^#^
BCR-ABL	14(3.5)	9(5.6)	5(2.1)	0.063^#^
MLL	23(5.8)	8(5.0)	15(6.3)	0.575
Cytogenetics				
Abnormal karyotype	209(52.4)	92(57.1)	117(49.2)	0.117
Complex karyotype	71(17.8)	26(16.1)	45(18.9)	0.480
Monosomy karyotype	44(11.0)	16(9.9)	28(11.8)	0.568
t8-21	23(5.8)	13(8.1)	10(4.2)	0.103
inv16	15(3.8)	6(3.7)	9(3.8)	0.977^#^
t9-22	5(1.3)	3(1.9)	2(0.8)	0.658^#^
plus8	38(9.5)	16(9.9)	22(9.2)	0.817
del5q	29(7.3)	14(8.7)	15(6.3)	0.366
del7	25(6.3)	9(5.6)	16(6.7)	0.647
del17p	14(3.5)	6(3.7)	8(3.4)	0.846^#^
Abnormal NT-proBNP	101(25.3)	58(36.0)	43(18.1)	< 0.001
Echocardiogram indexes				
LA (mm)	34(30–38)	34(31-37.5)	33(29.75-38)	0.357
LV (mm)	45(42–48)	45(43–48)	46(42–48)	0.871
IVS (mm)	10(9–11)	10(9–11)	10(9–11)	0.019
EDV (ml)	97(78–113)	95(80-108.5)	97(78–118)	0.254
ESV (ml)	31(25–40)	31(24–41)	30(25–39)	0.910
SV (ml)	66(55–78)	64(53–75)	67(56–80)	0.172
EF (%)	65(60–70)	64(60-70.5)	66(60–70)	0.062

#: Fisher’s exact test

NPM1: nucleophosmin 1; CEBPA: CCAAT/enhancer-binding protein alpha; FLT3: Fms-like tyrosine kinase 3; TP53, Tumor Protein P53; RUNX1: runt-related transcription factor 1; TET2: tet methylcytosine dioxygenase 2; IDH: Isocitrate dehydrogenase; WT1: Wilm tumor gene1; JAK2: Janus kinase 2; AML1-ETO: acute myeloid leukemia 1 Eight-twenty-one; CBFB-MYH11: core-binding factor, beta subunit-myosin heavy chain 11; BCR-ABL: breakpoint cluster region-Abelson; MLL: Mixed lineage leukemia; NT-pro BNP: N-terminal pro-B-type natriuretic peptide; WBC: White blood cells; RBC: Red blood cells; Hb: Hemoglobin; PLT: Platelets; LA: Left atrium; LV: Left ventricle; IVS: Interventricular septum; EDV: End-diastolic volume; ESV: End-systolic volume; SV: stroke volume; EF: Ejection fraction; BM: bone marrow

### Baseline characteristics of the training set and testing set

The data of 399 AML patients from Wuhan University Zhongnan Hospital were grouped into a training set and a testing set by the random digital grouping method, as shown in Table 2. Of the 279 patients in the training set, 113 (40.5%) had cardiac injury, and of the 120 patients in the testing set, 48 (40%) had cardiac injury. There were no statistical differences in baseline characteristics of the patients between the training and testing set (*P* > 0.05). In addition, of the 100 cases in the externally validated cohort, 48 were combined with cardiac injury. The comparison between AML patients with and without cardiac injury in the training set, testing set, and external validation set was detailed in Table 3.

**Table 2:** Comparisons of baseline characteristics between training set and testing set

Characteristics	Training set (n = 279) No. (%) / Median (IQR)	Testing set (n = 120) No. (%) / Median (IQR)	*P*
Cardiac injury	113(40.5)	48(40.0)	0.925
Gender			0.993
Male	151(54.1)	65(54.2)	
Female	128(45.9)	55(45.8)	
Age (years)			0.593
Age < 60	150(53.8)	68(56.7)	
Age ≥ 60	129(46.2)	52(43.3)	
Smoking history	47(16.8)	20(16.7)	0.965
History of hypertension	47(16.8)	20(16.7)	0.965
History of diabetes	21(7.5)	8(6.7)	0.761
WBC (10^9^/L)	10.34(2.23–40.62)	8.835(2.005–25.98)	0.395
RBC (10^12^/L)	2.2(1.7–2.66)	2.185(1.7–2.58)	0.827
Hb (g/L)	70(56.2–86.2)	69.85(56.725–84.45)	0.946
PLT (10^9^/L)	37(20–71)	40(21-81.5)	0.307
BM blast ratio (%)	59.5(35.5–81.5)	59.25(35.75–78.05)	0.740
Gene mutations			
NPM1	43(15.4)	20(16.7)	0.753
CEBPA	31(11.1)	11(9.2)	0.562
FLT3	47(16.8)	20(16.7)	0.965
C-kit	12(4.3)	3(2.5)	0.562
TP53	17(6.1)	7(5.8)	0.920
RUNX1	11(3.9)	6(5.0)	0.632
Ras	16(5.7)	4(3.3)	0.313
TET2	16(5.7)	10(8.3)	0.335
IDH	25(9.0)	12(10.0)	0.743
WT1	165(59.1)	70(58.3)	0.881
JAK2	6(2.2)	5(4.2)	0.427
AML1-ETO	19(6.8)	10(8.3)	0.591
CBFB-MYH11	13(4.7)	4(3.3)	0.548
BCR-ABL	9(3.2)	5(4.2)	0.864
MLL	14(5.0)	9(7.5)	0.329
Cytogenetics			
Abnormal karyotype	149(53.4)	60(50.0)	0.532
Complex karyotype	44(15.8)	27(22.5)	0.107
Monosomy karyotype	28(10.0)	16(13.3)	0.335
t8-21	15(5.4)	8(6.7)	0.612
inv16	12(4.3)	3(2.5)	0.562
t9-22	4(1.4)	1(0.8)	0.997
plus8	25(9.0)	13(10.8)	0.559
del5q	22(7.9)	7(5.8)	0.469
del7	17(6.1)	8(6.7)	0.828
del17p	9(3.2)	5(4.2)	0.864
Abnormal NT-proBNP	70(25.1)	31(25.8)	0.875
Echocardiogram indexes			
LA (mm)	34(30–38)	34(30–38)	0.823
LV (mm)	45(42–48)	46(42.25-48)	0.678
IVS (mm)	10(9–11)	10(9–11)	0.312
EDV (ml)	97(78–111)	97(82.25–113)	0.930
ESV (ml)	31(25–40)	30(24.25-41)	0.704
SV (ml)	64(54–77)	68(56-78.75)	0.256
EF (%)	65(60–70)	65.5(60–71)	0.839

NPM1: nucleophosmin 1; CEBPA: CCAAT/enhancer-binding protein alpha; FLT3: Fms-like tyrosine kinase 3; TP53, Tumor Protein P53; RUNX1: runt-related transcription factor 1; TET2: tet methylcytosine dioxygenase 2; IDH: Isocitrate dehydrogenase; WT1: Wilm tumor gene1; JAK2: Janus kinase 2; AML1-ETO: acute myeloid leukemia 1 Eight-twenty-one; CBFB-MYH11: core-binding factor, beta subunit-myosin heavy chain 11; BCR-ABL: breakpoint cluster region-Abelson; MLL: Mixed lineage leukemia; NT-pro BNP: N-terminal pro-B-type natriuretic peptide; WBC: White blood cells; RBC: Red blood cells; Hb: Hemoglobin; PLT: Platelets; LA: Left atrium; LV: Left ventricle; IVS: Interventricular septum; EDV: End-diastolic volume; ESV: End-systolic volume; SV: stroke volume; EF: Ejection fraction; BM: bone marrow

**Table 3:** Distribution and comparisons of AML patients with or without cardiac injury in the training set, testing set and external validation set

	Training set			Testing set			External validation set		
Characteristics	Cardiac injury (n = 113)	No cardiac injury (n = 166)	*P*	Cardiac injury (n = 48)	No cardiac injury (n = 72)	*P*	Cardiac injury (n = 48)	No cardiac injury (n = 52)	*P*
Gender			0.652			0.708			0.393
Male	63(55.8)	88(53.0)		25(52.1)	40(55.6)		29(60.4)	27(51.9)	
Female	50(44.2)	78(47.0)		23(47.9)	32(44.4)		19(39.6)	25(48.1)	
Age (years)			0.159			0.764			0.030
Age < 60	55(48.7)	95(57.2)		28(58.3)	40(55.6)		21(43.8)	34(65.4)	
Age ≥ 60	58(51.3)	71(42.8)		20(41.7)	32(44.4)		27(56.2)	18(34.6)	
Smoking history	20(17.7)	27(16.3)	0.753	7(14.6)	13(18.1)	0.617	14(29.2)	10(19.2)	0.245
History of hypertension	21(18.6)	26(15.7)	0.522	7(14.6)	13(18.1)	0.617	9(18.8)	6(11.5)	0.313
History of diabetes	13(11.5)	8(4.8)	0.038	3(6.2)	5(6.9)	1.000	6(12.5)	3(5.8)	0.409
WBC (10^9^/L)	27.56(6.53-76.505)	5.52(1.7675–16.065)	< 0.001	21.205(5.0675–86.14)	4.6950(1.6375–14.2175)	< 0.001	28.115(3.77-64.7125)	4.315(1.9-23.7675)	0.011
RBC (10^12^/L)	2.32(1.83–2.88)	2.14(1.6325-2.56)	0.018	2.295(1.84-2.8575)	2.07(1.655-2.5)	0.033	2.385(1.8075–2.945)	2.23(1.7975-2.56)	0.210
Hb (g/L)	70(58.65-87)	69.85(54.75-85.425)	0.331	70.85(59.775–90.375)	68.8(54.375–82.95)	0.263	78.4(61-88.7)	72.2(58.775–88.5)	0.558
PLT (10^9^/L)	31(17.5–70)	38(22-72.25)	0.357	39(22-89.5)	43.5(20.25-73)	0.682	29.5(22.25-96)	37.5(23.25–71.5)	0.959
BM blast ratio (%)	65.2(37.95–83.75)	56.15(31.875–77.35)	0.015	59.75(34.825-83)	57.75(36.625–72.875)	0.247	65.75(48.47-85.6675)	62.25(24.7775-79)	0.013
Gene mutations									
NPM1	29(25.7)	14(8.4)	< 0.001	13(27.1)	7(9.7)	0.012	14(29.2)	9(17.3)	0.159
CEBPA	13(11.5)	18(10.8)	0.863	4(8.3)	7(9.7)	1.000	10(20.8)	7(13.5)	0.327
FLT3	30(26.5)	17(10.2)	< 0.001	13(27.1)	7(9.7)	0.012	16(33.3)	9(17.3)	0.064
C-kit	7(6.2)	5(3.0)	0.324	2(4.2)	1(1.4)	0.720	4(8.3)	5(9.6)	1.000
TP53	8(7.1)	9(5.4)	0.570	4(8.3)	3(4.2)	0.578	5(10.4)	2(3.8)	0.371
RUNX1	4(3.5)	7(4.2)	1.000	3(6.2)	3(4.2)	0.932	4(8.3)	3(5.8)	0.913
Ras	10(8.8)	6(3.6)	0.065	3(6.2)	1(1.4)	0.350	5(10.4)	5(9.6)	1.000
TET2	7(6.2)	9(5.4)	0.785	7(14.6)	3(4.2)	0.092	7(14.6)	4(7.7)	0.271
IDH	12(10.6)	13(7.8)	0.423	6(12.5)	6(8.3)	0.664	5(10.4)	4(7.7)	0.900
WT1	77(68.1)	88(53.0)	0.012	32(66.7)	38(52.8)	0.131	22(45.8)	22(42.3)	0.723
JAK2	5(4.4)	1(0.6)	0.082	5(10.4)	0(0)	0.020	3(6.2)	1(1.9)	0.554
AML1-ETO	9(8.0)	10(6.0)	0.528	5(10.4)	5(6.9)	0.736	5(10.4)	0(0)	0.054
CBFB-MYH11	5(4.4)	8(4.8)	0.878	1(2.1)	3(4.2)	0.917	2(4.2)	4(7.7)	0.749
BCR-ABL	5(4.4)	4(2.4)	0.555	4(8.3)	1(1.4)	0.162	1(2.1)	2(3.8)	1.000
MLL	5(4.4)	9(5.4)	0.708	3(6.2)	6(8.3)	0.944	2(4.2)	2(3.8)	1.000
Cytogenetics									
Abnormal karyotype	66(58.4)	83(50.0)	0.167	26(54.2)	34(47.2)	0.456	31(64.6)	29(55.8)	0.369
Complex karyotype	16(14.2)	28(16.9)	0.542	10(20.8)	17(23.6)	0.721	9(18.8)	7(13.5)	0.471
Monosomy karyotype	11(9.7)	17(10.2)	0.890	5(10.4)	11(15.3)	0.443	7(14.6)	3(5.8)	0.257
t8-21	8(7.1)	7(4.2)	0.298	5(10.4)	3(4.2)	0.331	2(4.2)	0(0)	0.228
inv16	5(4.4)	7(4.2)	1.000	1(2.1)	2(2.8)	1.000	3(6.2)	4(7.7)	1.000
t9-22	2(1.8)	2(1.2)	1.000	1(2.1)	0(0)	0.400	1(2.1)	2(3.8)	1.000
plus8	10(8.8)	15(9.0)	0.957	6(12.5)	7(9.7)	0.631	4(8.3)	2(3.8)	0.601
del5q	11(9.7)	11(6.6)	0.344	3(6.2)	4(5.6)	1.000	5(10.4)	2(3.8)	0.371
del7	7(6.2)	10(6.0)	0.953	2(4.2)	6(8.3)	0.601	4(8.3)	3(5.8)	0.913
del17p	3(2.7)	6(3.6)	0.920	3(6.2)	2(2.8)	0.641	2(4.2)	3(5.8)	1.000
Abnormal NT-proBNP	41(36.3)	29(17.5)	< 0.001	17(35.4)	14(19.4)	0.050	21(43.8)	5(9.6)	< 0.001
Echocardiogram indexes									
LA (mm)	34(31–38)	33(29–38)	0.459	34.5(31.25-37)	34(30–38)	0.588	35.5(31–38)	33(29–39)	0.445
LV (mm)	45(43–48)	45(42–48)	0.700	46(41.5–48)	46(42.25-48)	0.778	43.5(38.25-49)	45(41–48)	0.663
IVS (mm)	10(9–11)	10(9–11)	0.070	10(9.25-12)	10(9–11)	0.125	10(9–12)	10(9–11)	0.371
EDV (ml)	95(83-107.5)	97(78–118)	0.471	93.5(78-110.5)	99(83–118)	0.314	101.5(87-118.25)	100(88-108.75)	0.942
ESV (ml)	32(25–41)	30(25–39)	0.404	30.5(22–44)	30(25–39)	0.271	35(28-43.25)	31(25.25-39)	0.168
SV (ml)	64(53–73)	67(55–80)	0.151	68.5(56-77.75)	67.5(56.75–82.25)	0.799	68(56.25-78)	68(55.5–79)	0.855
EF (%)	63(60–69)	66.5(60–70)	0.045	64.5(60–71)	66(59.25–71.75)	0.674	60(57–66)	65(61.25–70.75)	< 0.001

NPM1: nucleophosmin 1; CEBPA: CCAAT/enhancer-binding protein alpha; FLT3: Fms-like tyrosine kinase 3; TP53, Tumor Protein P53; RUNX1: runt-related transcription factor 1; TET2: tet methylcytosine dioxygenase 2; IDH: Isocitrate dehydrogenase; WT1: Wilm tumor gene1; JAK2: Janus kinase 2; AML1-ETO: acute myeloid leukemia 1 Eight-twenty-one; CBFB-MYH11: core-binding factor, beta subunit-myosin heavy chain 11; BCR-ABL: breakpoint cluster region-Abelson; MLL: Mixed lineage leukemia; NT-pro BNP: N-terminal pro-B-type natriuretic peptide; WBC: White blood cells; RBC: Red blood cells; Hb: Hemoglobin; PLT: Platelets; LA: Left atrium; LV: Left ventricle; IVS: Interventricular septum; EDV: End-diastolic volume; ESV: End-systolic volume; SV: stroke volume; EF: Ejection fraction; BM: bone marrow

### Risk factors and predictive models of cardiac injury

**Fig. 3:**
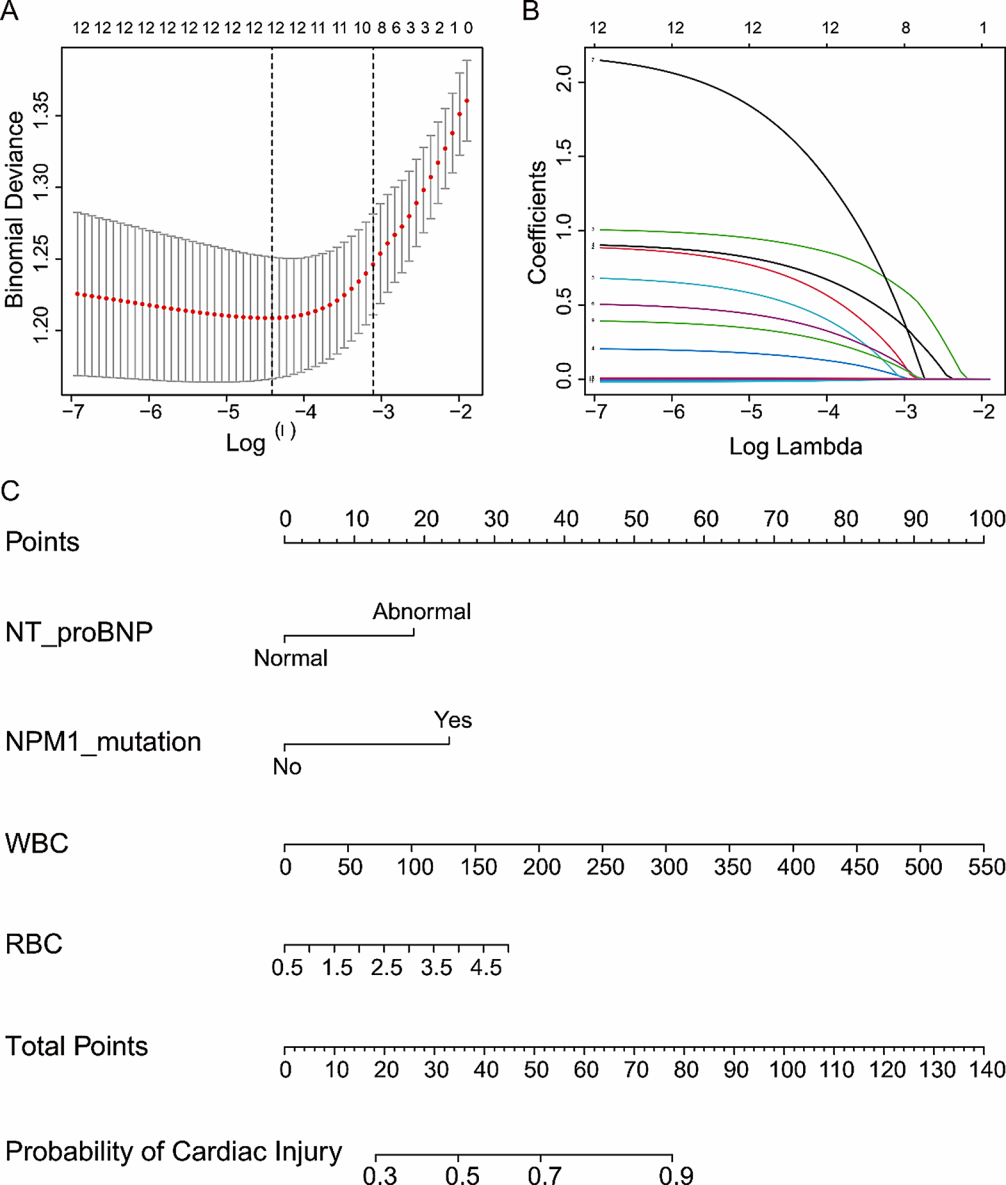
Screening of variables related to cardiac injury in AML patients and construction of a prediction model. Variables selection was performed using the LASSO regression model in the training set. (**A**) The selected 10 variables were analyzed by LASSO regression, and two dashed lines marked the best value. (**B**) LASSO coefficient profile for 12 variables and the standard gives ten nonzero coefficients. (**C**) Nomogram of the prediction model for cardiac injury in newly diagnosed AML patients in the training set. To estimate the risk of cardiac injury, the points for each variable were calculated by drawing a straight line from the patient’s variable value to the axis labeled “points”. The total points were converted to “probability of cardiac injury” on the lowest axis

In the training set, variables with *P* < 0.1 were screened by univariate logistic regression analysis. These 12 variables were analyzed by LASSO regression, and 10 non-zero coefficients in lambda.1se were selected as candidate variables. The lambda.1se corresponding to λ value of 0.0447 was chosen and ten predictors were selected the model (Fig. 3A-B). The coefficients for each parameter were as follows: 0.4029 for abnormal NT-pro BNP, 0.1706 for history of diabetes, 0.6376 for NPM1, 0.0294 for FLT3, 0.0383 for Ras, 0.1220 for WT1, 0.5206 for JAK2, 0.0055 for WBC, 0.0942 for RBC, and − 0.0007 for EF. Then, by multivariate analysis, abnormal NT-pro BNP, NPM1, WBC and RBC were still independent risk factors for cardiac injury, and the odds ratio (OR) of the four variables were shown in Table 4.

**Table 4:** Multivariate logistic regression analyses of cardiac injury in AML patients from training set

Variables	Coefficients	SE	OR	95%CI	P
History of diabetes					
No	Ref.		Ref.		
Yes	0.875133	0.529612	2.4	0.85–6.77	0.098
Genetic mutations					
NPM1					
No	Ref.		Ref.		
Yes	1.10079	0.39808	3.01	1.38–6.56	0.006
FLT3					
No	Ref.		Ref.		
Yes	0.240088	0.406053	1.27	0.57–2.82	0.554
Ras					
No	Ref.		Ref.		
Yes	0.678078	0.581091	1.97	0.63–6.15	0.243
WT1					
No	Ref.		Ref.		
Yes	0.487583	0.306741	1.63	0.89–2.97	0.112
JAK2					
No	Ref.		Ref.		
Yes	2.178846	1.139206	8.84	0.95–82.41	0.056
NT-proBNP					
Normal	Ref.		Ref.		
Abnormal	0.911099	0.340318	2.49	1.28–4.85	0.007
WBC (10^9^/L)	0.008208	0.002956	1.0082	1.0024–1.0141	0.005
RBC (10^12^/L)	0.376219	0.190353	1.46	1-2.12	0.048
EF (%)	-0.02026	0.018682	0.98	0.94–1.02	0.278

NPM1: nucleophosmin 1; FLT3: Fms-like tyrosine kinase 3; WT1: Wilm tumor gene1; JAK2: Janus kinase 2; NT-pro BNP: N-terminal pro-B-type natriuretic peptide; WBC: White blood cells; RBC: Red blood cells; EF: Ejection fraction

### Nomogram establishment and validation

Based on the above analysis results, abnormal NT-pro BNP, NPM1, WBC and RBC were included in the prediction model as independent risk factors. We generated a cardiac injury risk model and presented it in the form of a nomogram to visually illustrate the probability of cardiac injury in newly diagnosed AML patients (Fig. 3C).

**Fig. 4:**
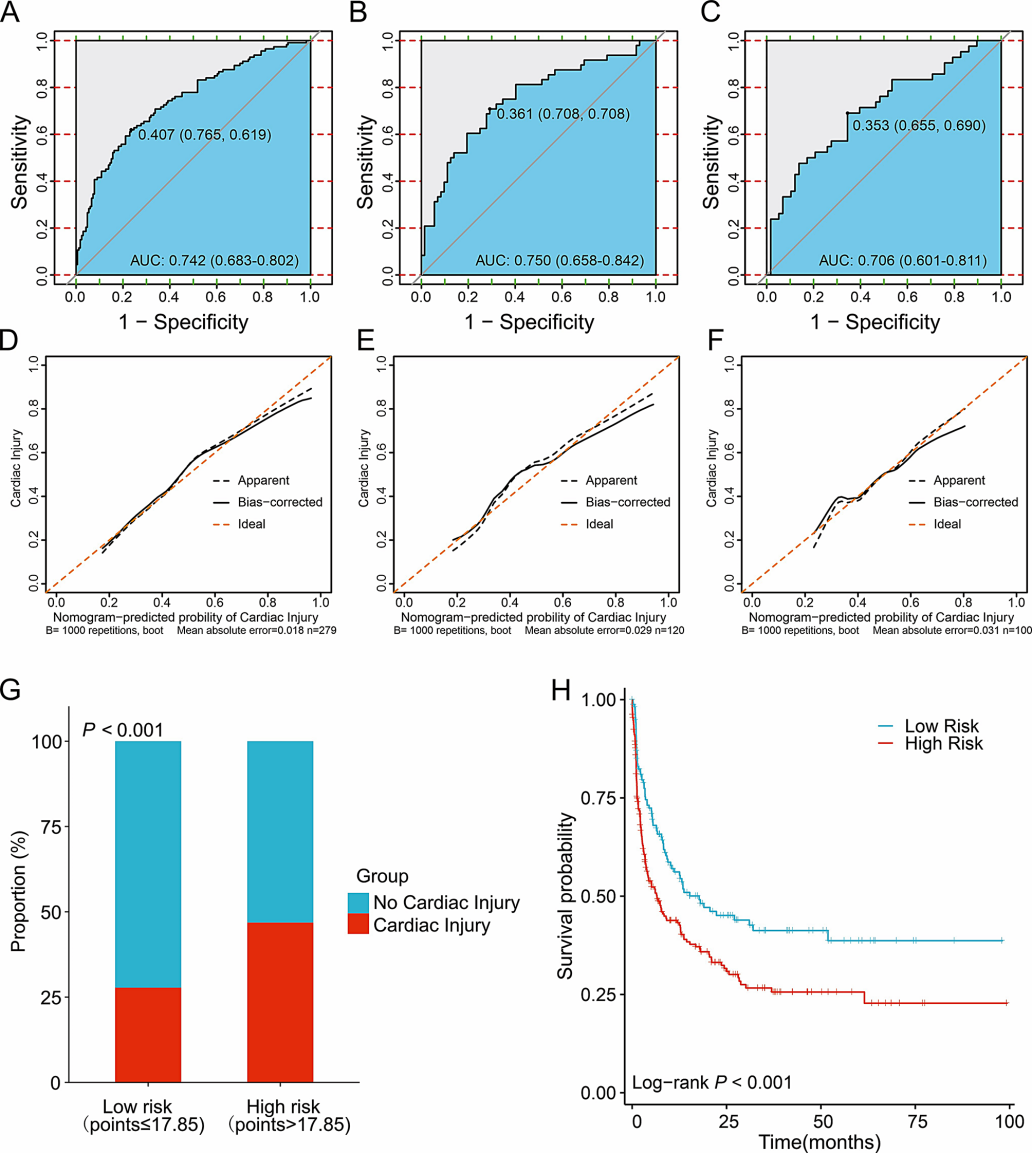
The performance of the scoring system to predict the probability of cardiac injury in AML patients. ROC curves and AUCs to evaluate the prediction accuracy in training set (**A**), testing set (**B**) and the external validation set (**C**). Calibration curves to assess the agreement of actual probabilities and predicted probabilities for prediction accuracy in the training set (**D**), testing set (**E**) and the external validation set (**F**). Cardiac injury predicting effects of the risk score in nomograms. Our nomogram divided participants into two subgroups according to the optimal threshold of ROC. The proportion of cardiac injury in each subgroup is in (**G**), *P* < 0.001. The OS was also assessed in different risk subgroups with *P* < 0.001 (**H**). ROC, receiver operating characteristic; AUC, area under the ROC curve. OS, overall survival

By using ROC analysis, the model showed high diagnostic accuracy in the training set, testing set, and external validation set (Fig. 4A-C). Our model had an AUC of 0.742 (0.683–0.802) on the training set. Meanwhile, the AUCs in the testing set and external validation set were 0.750 (0.658–0.842) and 0.706 (0.601–0.811), respectively, showing good prediction accuracy. The numbers on the subfigures represent the Youden index and its corresponding specificity and sensitivity. The performance of the model was validated by calibration curves, and no significant deviation from the reference line was observed on the training, testing, and external validation sets (after repetitive Bootstrap self-sampling 1,000 times show that the mean absolute errors of the simulated curves and the actual curves were 0.018, 0.029, and 0.031, respectively), suggesting good consistency between predictions in the training set and actual observations (Fig. 4D-F).

### Risk score in Nomogram

We calculated nomogram scores for all newly diagnosed AML patients using R. Here, we showed all scores for each variable in a nomogram (Table 5). Patients were divided into low risk (points ≤ 17.85) and high risk (points > 17.85) according to the optimal threshold of the training set ROC curve. We found that the degree of cardiac injury in newly diagnosed AML patients increased with increasing risk (*P* < 0.001). KM curves were applied to better visualize the relationship between risk stratification and survival prognosis, which indicated that AML patients with higher risk of cardiac injury had a worse prognosis (*P* = 0.001, Fig. 4G-H).

**Table 5:** Points given for each variable in the nomogram

Variables	Nomogram points
NT-proBNP	
Abnormal	18
Normal	0
NPM1-mutation	
Yes	24
No	0
WBC (10^9^/L)	
0	0
50	9
100	18
150	27
200	36
250	45
300	55
350	64
400	73
450	82
500	91
550	100
RBC (10^12^/L)	
0.5	0
1	4
1.5	7
2	11
2.5	14
3	18
3.5	21
4	25
4.5	28
5	32

## Discussion

In this study, we developed and validated a personalized predictive nomogram and risk score for the risk of cardiac injury before chemotherapy in patients with newly diagnosed AML, using cost-effective and readily available variables to help hematologists identify high-risk patients. The prediction model includes four variables: WBC, RBC, NT-proBNP, and NPM1 mutations. Internal and external validation showed that our nomogram and risk scores have good predictive performance.

In recent years, the field of cardio-oncology has made significant progress in elucidating the intricate relationship between tumors and the heart. While much attention has been devoted to studying cardiac injury resulting from anti-tumor therapy, injury caused by the tumor itself is also coming into the limelight [25]. A large Danish HF Cohort study reported that when diagnosed with cancer, their all-cause mortality rate was higher than that of without HF [26]. Xiao W et al. found that newly diagnosed AL patients did experience some cardiac-related lesions prior to chemotherapy, and this injury persisted after adjusting for confounding factors. In addition, cardiac enzyme abnormalities were more severe in the hyperleukocytic leukemia subgroup (WBC count ≥ 100 × 10^9^/L), and the proportion of blasts was positively correlated with cardiac injury [20]. In agreement with our result, a retrospective study from Harvard Medical School also found that AL was associated with cardiac alterations before chemotherapy [27], which together with other similar studies [28, 29], suggests that leukemia itself may cause direct damage to the heart prior to anti-tumor therapy.

From an etiological perspective, several factors may explain the development of cardiac injury in AL patients before chemotherapy. First, these two entities have overlapping risk factors, such as hypertension, diabetes, genetic risk factors, etc. [30]. Second, both share common systemic pathogenic pathways and mechanisms, such as abnormal production of pro-inflammatory cytokines, metabolic reprogramming in the tumor environment, clonal hematopoiesis of uncertain potential, crosstalk between stromal cells and the extracellular environment, etc. [8]. Finally, the direct infiltration of leukemic cells into the heart has been identified as another potential mechanism [11, 12, 31–33]. Additionally, the investigation of cytokine networks in AML has gained increasing attention in recent years. Some pro-inflammatory mediators such as IL-1β, TNF-α and IL-6 tend to increase AML aggressiveness and can promote the survival and proliferation of AML cells [34]. And these can also cause heart damage [35, 36].

As previously stated, the bidirectional relationship between AML and cardiac injury status makes the diagnosis of cardiac injury potentially rather delayed, in part because symptoms of the former are attributable to the latter and vice versa [37]. Certain studies have suggested that the assessment of global longitudinal strain peak during systole using speckle tracking echocardiography may sensitively monitor early cardiotoxic alterations [38, 39]. However, this approach is limited to hospitals equipped with the necessary resources. Furthermore, cardiac biomarkers, including troponin, BNP, and cardiac enzymes [40] or other potential biomarkers such as glycogen phosphorylase BB [41], total antioxidant status, circulating microRNAs [42] and endothelial dysfunction [43] should be comprehensively assessed. The complexity and specialization involved make it easy for non-cardiac doctors to miss tests when focusing on the patient’s pre-treatment cardiac status and therefore slightly less clinically implementable.

We developed and validated a nomogram of cardiac injury risk before chemotherapy in patients with newly diagnosed AML. The model demonstrated good predictive performance. Nomograms combine several modeling algorithms to calculate continuous probabilities for specific outcomes. Among the many predictive tools currently available, nomograms have higher accuracy and better discriminative features [44]. We used LASSO regression and the multivariate logistic regression in the screening process, considering the collinearity and interaction of the screened variables. Besides, we performed a complete evaluation of the model for discrimination, clinical use and calibration, as well as an external validation of the model. Moreover, for further convenience of clinical use, we also established a risk score, and risk stratification was performed.

In clinical practice, AML patients are applied chemotherapy regimens very quickly after being definitively diagnosed, which often directly exacerbates the occurrence of adverse cardiac effects [19]. Generally, we attribute these cardiac injuries to the treatment, ignoring the heart’s injury state. Therefore, it is essential to promptly recognize the risk of cardiac injury in patients with incipient AML. In this study, we provide a prediction model that can help clinicians identify patients with incipient AML at high risk of cardiac injury and help them choose more appropriate treatment options promptly. Our nomogram model and risk score are routine clinical variables that are readily available to hematology clinicians, and as such, they can be easily applied in practice.

Our model includes four indicators: NT-proBNP, NPM1 mutations, WBC, and RBC. The level of NT-proBNP was closely related to the state of the heart [45]. AML with NPM1 mutations, although considered to have a good prognosis, results in low patient survival and high relapse rates due to its frequent coexistence with FLT3-ITD [46]. In addition, it often combines to exhibit higher leukocyte levels, percentage of BM blasts, cell invasiveness, and an incidence of extramedullary involvement [47]. AML patients with leukocytosis were prone to vascular stagnation and accumulation. In addition, the increase in metabolic byproducts in the state of blast cells will also increase the risk of cardiac injury [48]. The erythropoietic activity of AML patients was more active, especially those with BM involvement [49]. Furthermore, under certain conditions of excess oxidative stress, erythrocytes may switch the redox balance, promoting oxidative stress and being detrimental to resident and circulating adjacent cell types [50]. The latest research found that RBCs may be carriers of cytokines, which can induce significant damage to endothelial function, thereby causing vascular dysfunction [51]. This provides a new idea for cardiac injury in AML state.

Although we have developed a predictive model for the risk of cardiac injury before chemotherapy in patients with newly diagnosed AML patients, and tried to predict the risk with a minimum number of markers, this real-world study still has certain limitations. First, although hs-TNI is a more sensitive indicator of the degree of myocardial damage, it was combined with the diagnostic value of CK-MB in order to investigate this scientific question as it is not routinely examined in AML patients. Second, we used multiple imputations to replace missing values. This has the potential to lead to bias. Third, compared with other published disease prediction models, nomogram makes it difficult to deal with some nonlinear effects in the algorithm [52–54]. In the future, we can consider collaborating with other professionals to optimizing the model algorithm and verifying the model effect with multiple calibrations to screen the optimal model. Fourth, our sample size is small and focuses on Asian people, which affects the extrapolation of the model to some extent. Although there is much room for improvement in this study, we believe that as cardiac oncology develops, this interdisciplinary model of collaboration will eventually bridge the gap between the two fields. It still needs multi-center, large-sample clinical studies or other related work to test it before it can be widely accepted or applied.

## Conclusion

We developed and validated a personalized predictive nomogram for cardiac injury risk before chemotherapy in patients with newly diagnosed AML. The nomogram has good predictability and generalizability. It can provide individualized risk assessment for patients and help clinicians manage related decisions. In addition, substantial clinical and other related work is required before this nomogram can be widely accepted and used.

## Data Availability

All data are available upon reasonable request from the corresponding author. The authors will unreservedly provide raw data supporting the conclusions of this paper.
